# An Intelligent Parking Management System for Urban Areas

**DOI:** 10.3390/s16060931

**Published:** 2016-06-21

**Authors:** Juan A. Vera-Gómez, Alexis Quesada-Arencibia, Carmelo R. García, Raúl Suárez Moreno, Fernando Guerra Hernández

**Affiliations:** 1 Edosoft Factory, S.L. Las Palmas de Gran Canaria, Las Palmas de Gran Canaria 35011, Spain; juan.vera@edosoft.es (J.A.V.-G.); raul.suarez@edosoft.es (R.S.M.); fernando.guerra@edosoft.es (F.G.H.); 2 Instituto Universitario de Ciencias y Tecnologías Cibernéticas, Universidad de Las Palmas de Gran Canaria, Las Palmas de Gran Canaria 35017, Spain; rgarcia@dis.ulpgc.es

**Keywords:** intelligent transport systems, parking, wireless sensor network (WSN), traffic, sensor, infrared

## Abstract

In this article we describe a low-cost, minimally-intrusive system for the efficient management of parking spaces on both public roads and controlled zones. This system is based on wireless networks of photoelectric sensors that are deployed on the access roads into and out of these areas. The sensors detect the passage of vehicles on these roads and communicate this information to a data centre, thus making it possible to know the number of vehicles in the controlled zone and the occupancy levels in real-time. This information may be communicated to drivers to facilitate their search for a parking space and to authorities so that they may take steps to control traffic when congestion is detected.

## 1. Introduction

Human mobility is a necessity in today’s world. It has a significant impact on both quality of life and the economy of modern societies. Transport systems are, therefore, a key element in developed or developing countries. Zhang [1], in his survey of Intelligent Transportation Systems, indicated that 40% of the world’s population spend at least one hour on the road every day. Of all the different modes of transport, the one that is used on a massive scale is land transport by road. Such large-scale use of this type of transport has led to congestion problems in densely populated metropolitan areas, with all the concomitant negative consequences. According to Shawe-Taylor [2], these negative consequences include pollution and its harmful effects on the environment and human health. Too much time on the road means an increase in energy consumption, which has a negative impact on both individual and national economies, as well as on the environment. Several medical studies [3] have confirmed that road transport congestion results in a deterioration of public health because it increases the risk of heart and respiratory diseases. Moreover, according to the WHO, over 7 million people die every year from health problems caused by pollution.

One of the causes of this excessive amount of time spent on the road in private road transport is the need to spend time looking for free parking spaces. Pineda [4] studied the costs generated by the extra distance vehicles have to travel to find a parking space in the cities of Madrid and Barcelona. The costs in consumption for the extra distance and time spent on the road are approximately 347 million and 268 million euros per year, respectively. Public transport authorities and the operators of parking spaces are evaluating various solutions to improve parking space management. Solutions based on infrastructure investment are expensive and implementation is slow. Technology-based solutions have been proposed as an alternative with lower costs and faster implementation.

In this article we describe a low-cost, minimally-intrusive system for the efficient management of parking spaces on both public roads and controlled zones. This system is based on wireless networks of photoelectric sensors that are deployed on the access roads into and out of these areas. The sensors detect the passage of vehicles on these roads and communicate this information to a data centre, thus making it possible to know the number of vehicles in the controlled zone and the occupancy levels in real-time. This information may be communicated to drivers to facilitate their search for a parking space and to authorities so that they may take steps to control traffic when congestion is detected.

The article is structured as follows: a selection of related studies is described in the second section, to provide context for our proposed system. The third section describes the system, explaining its general architecture, its main constituent elements, and the method developed to detect the passage of vehicles. Tests to verify system operation are described in the fourth section and, finally, the main conclusions are presented in section five.

## 2. Related Studies

Various authors have looked at developing sensor-based technological solutions to improve the use of parking spaces. According to Bagula [5], intelligent vehicle parking space management systems may be classified according to the type of sensor detection. He distinguishes the systems that only monitor the entry or exit of vehicles from the parking area from the systems that are able to detect whether each parking space is occupied or free. Systems belonging to the first type are easier to deploy and less expensive, appropriate for monitoring the occupancy levels of large outdoor parking areas. Systems belonging to the second type provide more useful and more detailed information to users and may be combined with positioning and guidance services to help locate the available spaces. This type of system is used in indoor parking spaces and is more complex and expensive than the entry and exit monitoring systems, as it requires that each parking space is equipped with sensors and a more sophisticated communications infrastructure. Various parking space management system proposals are described below.

Tang [6] proposed a wireless sensor network deployed in indoor car parks that shows the occupancy status of each parking space. Motes (sensor nodes) equipped with acoustic and light sensors are located in each space, and periodically notify whether the space is occupied or available. Benson [7] also proposed a network-based wireless sensor system. A communication link is established by ZigBee and the electromagnetic sensors were developed specifically for this system. Lin [8] proposed a vision-based parking management system to manage an outdoor car park using cameras set up around the parking space, sending information, including real-time display, to the ITS centre database. A scientific solution based on a GPS-based vehicle navigation system and the past and current status of the car park was proposed by Pullola [9], who modelled the availability of a car park using the Poisson process. The author also proposed an intelligent algorithm which helps the driver choose the parking space with the highest probability of being vacant. Lee [10] proposed a combination of magnetic and ultrasonic sensors to control car parks. This system is based on a modified version of the min-max algorithm for detection of vehicles using magnetometers and an algorithm for ultrasonic sensors. Srikanth [11] proposed an intelligent parking management system, consisting of a wireless network that uses different types of sensors to detect the presence of a vehicle in every one of the parking spaces; moreover, the system informs users and guides them to the location of the available space. The network’s sensor nodes communicate by radio frequency. Yoo [12] described a system, called S3, which is deployed in school zones, which is designed to detect and register vehicles driving at excessive speeds or parked in prohibited zones. This system consists of a wireless sensor network that is divided into two subnetworks: one to detect vehicles parked in prohibited zones and the other to detect vehicles travelling at excessive speeds. The sensors used are Anisotropic Magneto-Resistive (AMR) magnetic sensors, and the wireless communication link is established by ZigBee. Magrini [13] proposed a vision sensors network to monitor available spaces in public car parks, using distributed network nodes to perform the required processing and analysis of images. Chen [14] proposed a system for locating available spaces in indoor car parks and a guidance system to locate the available space. The architecture of this system is based on a wireless network of ultrasound sensors that detect the presence of a vehicle in each of the parking spaces. The status of each space is transmitted by a sensor node that sends this information via RFID to special routing nodes, which communicate with each other to relay the data packets sent by the sensor nodes to the control centre. The network has a tree topology. Assistance in locating the space is provided by LEDs that indicate the status of the parking space. In the context of public parking space management based on image sensors, Salvadori [15] described image-processing techniques, using threshold algorithms, to monitor parking spaces, achieving high-performance detection of their occupancy status. Alessanderlli [16] proposed the ScanTraffic architecture, which is used to develop a system to monitor traffic flow and parking spaces at the International Airport of Pisa; this system is also based on a wireless image sensor network. Liu [17] described the iParking system, which is designed to facilitate the location of available spaces in an indoor car park. The system receives data from sensors located inside the car park, which indicate the spaces that are free, and is able to guide the driver to a free space using a positioning system for indoor spaces that is based on smartphone capabilities. Gu [18] proposed a system for managing parking spaces on public roads. The system is called Street Parking System (SPS), and uses a three-axis magnetic sensor to detect vehicles and ZigBee technology for wireless communications, achieving a reliability rate close to 99% in vehicle detection. Reve [19] also proposed a similar system, based on a wireless sensor network and LED display system that indicates the available spaces to drivers at the car park access points. The sensors are infrared and communications are established by radio frequency (RF). In the context of the Smart City paradigm, Giuffrè [20] proposed an IPA (Intelligent Parking Assistant) conceptual architecture that aims to overcome current parking management problems. Yang [21] presented a prototype Smart Parking Services system, based on Wireless Sensor Networks (WSNs), for finding free parking spaces. The proposed scheme consists of wireless sensor networks, an embedded web server, a central web server, and a mobile phone application. Each parking space has a light sensor node that detects the status of the parking space, reporting periodically to the embedded web server via the wireless sensor networks. Using a Wi-Fi network, this information is sent to the central web server in real time, displaying the status of the parking spaces on the driver’s mobile device. Geng [22] proposed a smart parking system for urban areas. The system’s functions include parking detection, reservation guarantee and Vehicle-to-Infrastructure (V2I) or Infrastructure-to-Vehicle (I2V) communication. Considering the requirements of the user, the system assigns and reserves an optimal parking space combining proximity to destination and parking cost, ensuring that the overall parking capacity is efficiently utilised. The system was tested in a garage of Boston University. Tian [23] proposed an intelligent parking management system based on License Plate Recognition (LPR), which recognises the licence plate automatically at the car park access point and provides vehicle information; experimental results show that this parking management system can achieve 95% accuracy, and can be applied to real-time implementation. Karunamoorthy [24] proposed an intelligent parking system that uses image-processing techniques to solve the problem of unnecessary time consumption in finding a parking space in commercial car parks. This parking management system provides information about the available parking spaces, as well as an automated payment system for registered users. Caballero-Gil [25] proposed a low-cost service to predict and manage indoor parking spaces. This service is based on a central system that predicts the available spaces in the car park using cellular automata and an application for smartphones that uses various technologies to help drivers find free parking spaces.

In summary, this review of related studies shows that most of the proposed systems are designed to be deployed in indoor car parks and to monitor individual parking spaces, and that they usually include guidance services to locate the available space. From the point of view of the type of sensors used, these systems mainly use infrared and electromagnetic sensors. As for wireless communications, they use radio frequency, with ZigBee technology being the most used. In terms of network topology the most widely used is a tree topology.

The system proposed in this article is designed to monitor the entry and exit of vehicles into and out of outdoor parking zones located on public roads, not to monitor every single car space available, thus reducing the costs related to the deployment of technology. The system has been tested in closed car parks. On public roads, the system would not account for every single slot, but it is designed to provide estimations on real-time occupation levels. From a technological point of view, it uses a wireless network of photoelectric sensors, and uses 6LoWPAN over IEEE 802.15.4 for communications. It can be used across multiple communications platforms, using the IPv6 stack, which is hugely important to enable the IoT. IPv6 provides a basic transport mechanism to produce complex control systems and to communicate with other devices in a cost-effective manner using low-power wireless network. The network topology adopted for this system is a tree topology. As a result of mass production, the sensors used for the system have a very low cost in comparison with other sensors used for parking management systems, such as electromagnetic or pressure sensors. Despite their low cost, these sensors perform very well in detection applications; indeed, infrared sensors are widely employed in industrial automation processes, having proven reliability. Furthermore, their compact size makes it possible to enclose the whole node in a small box. In addition to this advantage, both the sensors themselves and the controlling boards have very low power consumption, and for this reason each sensor node is to be powered by a combination of batteries and solar power, minimising the deployment efforts and costs, as no wiring is needed. Another advantage of this approach over others is that it does not rely on the user’s involvement for the system to meet its purpose. There is no need for the vehicles to be equipped with smart location systems, or for the users to employ technological gadgets to access or update information about car park availability. The system is non-intrusive too: it does not identify the vehicles or their drivers at all, nor does it have to record or take pictures of them. Finally, this solution does not need more data processing than that done by the mainboards, further reducing infrastructure and system costs. Although it has not yet been developed, the system is intended to share parking availability data with the public authorities, contributing to a reduction in both traffic and its carbon footprint. In conclusion, the system, as will be seen in this article, is characterised by its ease of deployment, flexibility, and affordability when incorporating new nodes to monitor the access roads to the parking areas, and even new sensor subnets to monitor new parking areas.

## 3. Description of the System

To monitor the number of available spaces in the parking zones a low-cost, low-energy WSN will be employed. This means that a complex roll-out or new cabling infrastructure will not be required in the zone where the proposed system is deployed. The sensors will detect the passage of vehicles on the roads into and out of the parking areas located on public roads in real-time. The authorities and drivers will, therefore, know the occupancy levels of the parking zones at all times. The real-time response is essential because the system would not be practicable if there was a considerable delay in updating these data. Relaying pseudo real-time information to drivers avoids them having to spend more time on the road searching for a parking space, which hinders the circulation of other vehicles and increases fuel consumption, emissions, and noise pollution. Therefore, the proposed system will enable transport regulators to manage public parking zones sustainably. In addition, to facilitate a scalable and flexible roll-out in large urban areas, the system can make use of virtualisation and computing paradigms to obtain the processing and storage resources required by the system architecture. This will enable comprehensive, centralised handling of the data provided by the sensor network in a control centre.

**Figure 1:**
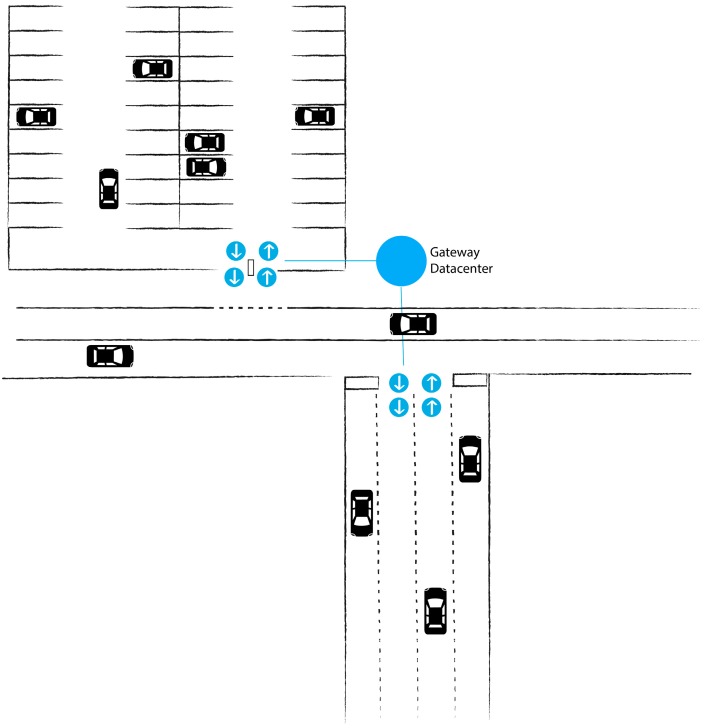
System architecture in a closed car park and on a public road.

Figure 1 shows the system being deployed on a public road and in a controlled parking area. The scheme represents the sensors and their direction on the access points and their communication with the gateway that relays the signal to the data centre. This gateway system may also provide in situ information on the occupancy level of both areas through an LED system.

### 3.1. System Architecture

The proposal consists of a network of wireless sensors located on roads in and out of public parking areas. These sensors detect the entry and exit of vehicles into and out of the parking areas, and transmit this information via the network. The sensors are connected to network nodes that receive the data provided by the sensors and transmit them across the network; this type of node is called a sensor node. In the network topology there is a node that enables the exchange of data between the sensor nodes and the data centre; this node is called a gateway node. Data obtained from the sensor network are stored and managed in the data centre, which is also where the operation of each sensor node is controlled. The data obtained by the data centre enable the provision of services that report on the occupancy levels of the parking spaces on public roads.

Each sensor node on the network will be developed on a proprietary platform that will be responsible for collecting the data obtained by the sensor or sensors associated with that node, interpreting and packaging information and transmitting it over the network. The sensor nodes must have low consumption levels, although when placed in outdoor parking areas it will be possible to add a small photovoltaic panel.

The gateway node links the sensor nodes and the data centre. The sensor nodes notify the data centre when a vehicle enters or exits the monitored parking area and receives orders that development or maintenance engineers wish to transmit to the network from the data centre, via the gateway.

The data centre controls the sensor network and manages the data provided by it. To carry out these procedures, this component of the architecture has two subsystems: the sensor network control subsystem that allows technical staff to configure and supervise the operations of the sensor network; and the subsystem for recording data provided by the sensors, which stores and processes the data in order to provide information services to users (authorities and drivers) about the status of the parking areas monitored by the system. To achieve scalability and flexibility in the data centre computing and storage resources, we propose using cloud virtualisation and computing paradigms in the system.

#### 3.1.1. Sensor Nodes

The sensor nodes send data packets that notify the entry or exit of a vehicle into or out of the monitored parking area. These packets include the identification number of the network and of the node to identify the parking area in question. Each sensor node interprets the messages received from the data centre. These packets contain the same fields as those sent by the node, but the identification fields of the network and node will correspond in this case to the terminal or terminals to which the message is directed, and the data field will contain the order sent by the data centre to the sensor nodes.

For the hardware components of the nodes two circuit boards were used: a mainboard and a sensor board. The mainboard includes the components responsible for data processing and communication between nodes, while the sensor board is connected to the sensor. These two circuit boards, the batteries and a solar panel are housed in a box with IP67 protection measuring 82 mm × 80 mm × 85 mm. The batteries are lithium polymer 1900 mAh, with a nominal voltage of 3.7 V, and the solar panel has a power output of 0.4 W (5 V/80 mA).

The main component of the mainboard is the CC2530 system-on-chip from Texas Instruments [26], a system that includes a microcontroller and a radio module in the integrated circuit. The microcontroller is the 16-bit Intel 8051 and has 8 KB of RAM and a programmable flash memory of 256 KB. The radio module is a transceiver that operates in the 2.4 GHz band and is compatible with the IEEE 802.15.4 protocol, with programmable output power of up to 4.5 dBm, allowing a maximum transmission speed of 250 Kbps. Another component that has been integrated into the mainboard is the CC2591 amplifier, also manufactured by Texas Instruments, which is compatible with the proposed radio system because it also operates in the 2.4 GHz band. This amplifier extends the range of communications by providing a power amplifier that increases output power and improves receiver sensitivity.

One of the main characteristics of the mainboard is its small size (50 × 70 mm). Wireless communication is established using the 6LoWPAN standard that enables the use of IPv6 over the IEEE 802.15.4 standard. The main objective of using this standard is to be able to use IPv6 on a low-energy WSN, thereby enabling small devices, with limited processing power, to achieve broad connectivity (interoperability) and to maximise the battery life (Mulligan [27]).

The mainboard also has an RS-232 interface, JTAG, and a number of expansion connectors allowing communication with external devices.

**Figure 2:**
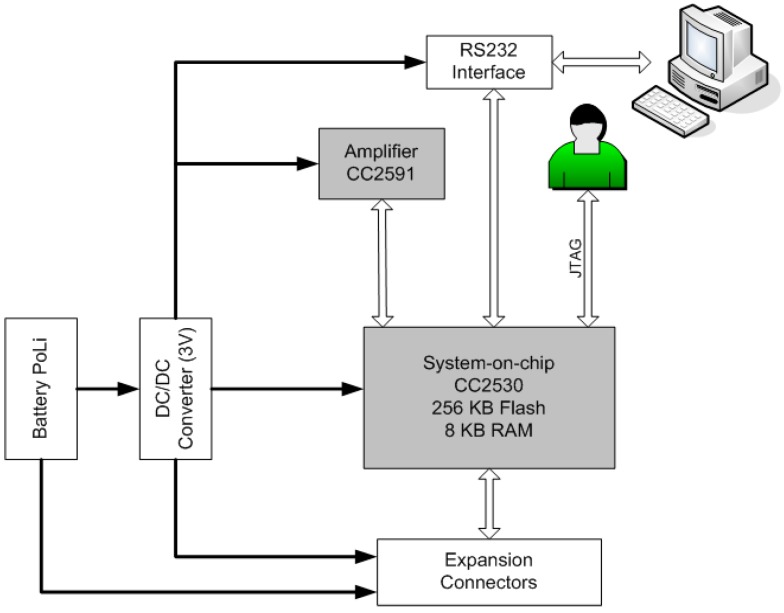
Block diagram of the mainboard.

As shown in Figure 2, in addition to these devices, a 3 V DC/DC converter has been added to the mainboard to adapt the voltage supplied by the battery to that required by the integrated circuits. This is necessary because the nominal battery voltage is 3.7 V.

**Figure 3:**
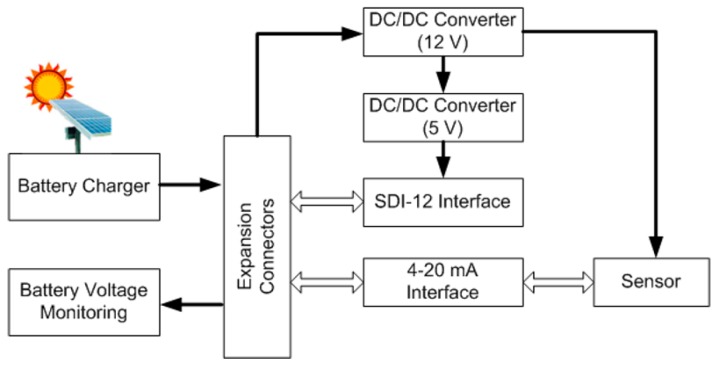
Block diagram of the sensor board.

As shown in Figure 3, the sensor board is composed of a series of interfaces and voltage converters to allow connection to a wide range of sensors. It has two DC/DC voltage converters, 12 V and 5 V, an SDI-12, and a 4–20 mA interface. This circuit board also features a battery charging system through an external solar panel, thus increasing the autonomy of the nodes.

**Figure 4:**
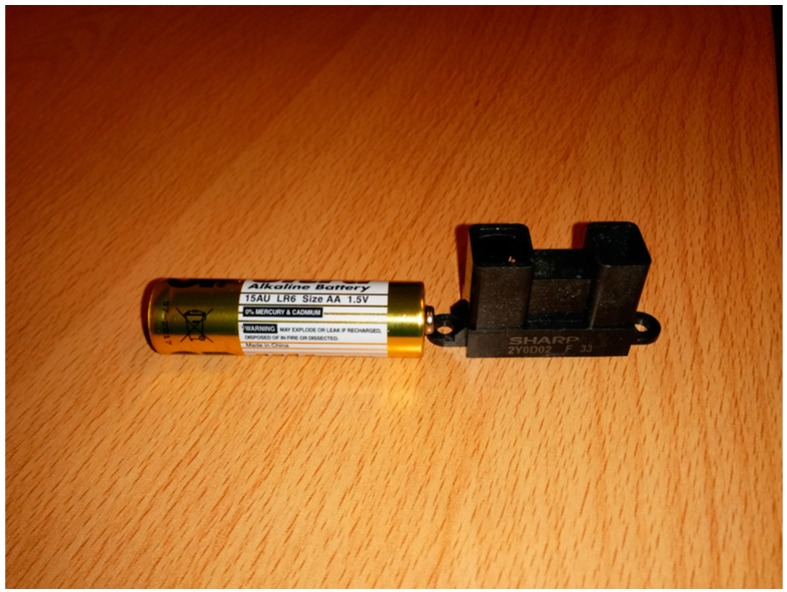
SHARP-GP2Y0D02YK0F sensor alongside a standard AA battery to give an idea of its size.

The photoelectric sensor used is a SHARP-GP2Y0D02YK0F (Sharp Corporation, Osaka, Japan) [28], shown in Figure 4.

This photoelectric sensor allows detection by the three types of standard sensing modes listed in Table 1.

The chosen sensing mode is the diffuse mode, justified by its simple installation requirements, with no calibration needed, and its detection performance. During the experimental process, sensors were deployed in speed bumps. However, future deployments could be altered and make use of street furniture like bollards, street lights, or traffic lights. When those are not available, the most straightforward installation is to deploy sensors on the ground, avoiding the need for retro-reflective or through-beam sensors.

**Figure 5:**
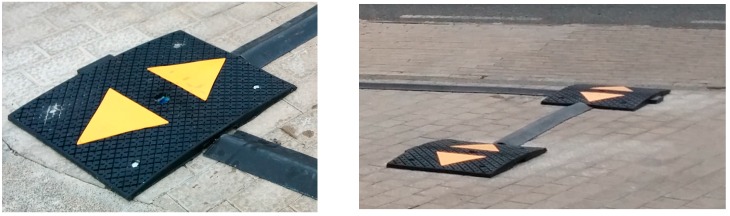
Installation of the sensors in two speed bumps (**left**) photoelectric sensor located at the centre of the speed bump; (**Right**) Pair of speed bumps with the photoelectric sensors.

To count vehicles entering and leaving the car park, two photoelectric sensors were used to avoid false positives. If a person or an object other than a vehicle interrupts the sensor IR beam, it could be incorrectly counted, but using two sensors and debugged controlling software, the count will not increase unless the pair of sensors actually detect a vehicle. This pair of sensors is fitted on speed bumps located on the entry and exit roads to the parking zones, as shown in Figure 5.

To develop the software for the sensor nodes we used the Contiki [29] operating system and the specific library for the CC2530 system-on-chip. Contiki is an open-source operating system geared towards the Internet of Things. It enables communication with microcontrollers via the Internet and can run on a wide range of wireless low-power devices, providing mechanisms to estimate the total energy consumption of the system and to identify the units that consume the most. In addition, Contiki is designed for small systems with only a few kilobytes of memory available; it is very efficient at memory management and provides different memory allocation mechanisms. With regard to communications, Contiki is fully compatible with the IPv6 and IPv4 standards, providing a full IP network stack, with standard protocols such as User Datagram Protocol (UDP), Transmission Control Protocol (TCP), and Hypertext Transfer Protocol (HTTP). Furthermore, it also supports low-power wireless communications on the 6LoWPAN, Routing Protocol for Low-Power and Lossy Networks (RPL) and CoAP standards.

#### 3.1.2. Gateway

The gateway node receives the packets sent by the sensor nodes and sends them on to the data centre. It also links communications in the opposite direction. From the hardware point of view, this element is composed of a mainboard similar to that used for the sensor nodes. Therefore, the gateway software will have characteristics and functionalities similar to that used for the sensor nodes without having to perform data packing or unpacking.

The gateway will communicate with the sensor nodes using the 6LoWPAN communication protocol. If there is a node in the data centre acting as a border router, communication between the base station and the gateway will be done by 6LoWPAN. However, if the gateway is located very close to the data centre, a RS-232 serial connection will be used.

#### 3.1.3. Data Centre

The data centre will receive information transmitted by the gateway and from the sensor nodes of the network, giving readings which indicate the passage of vehicles. It will also send the relevant instructions to the sensor network and store system data.

A server component will be installed to read and send data to the wireless sensor network while, for storing system information, a registration component will be used in the data centre.

The server component for reading and sending data to the sensor network was developed as a web service, using Play Framework with Java technology. This framework is inspired by Ruby on Rails and Django, and enables flexible web applications to be built. It is a modular framework with integrated unit testing (it includes support for JUnit and Selenium) that allows static methods, non-blocking asynchronous communication, and simple corrective maintenance.

The registration component consists of a database which will store information on registered users, the sensor networks deployed, the number of parking spaces available, *etc*. Specifically, we used PostgreSQL to set up the database. PostgreSQL functions include transactions, referential integrity, views and a multitude of features. It also incorporates Multiversion Currency Control—MVCC—which allows other clients to access it while a process is being written in a table without the need for locking. These features are suitable for systems that need to handle large amounts of data and a high number of concurrent users accessing the system simultaneously, as will be required by the proposed system.

### 3.2. Communications System

Communication between the sensor nodes and the gateway node will be performed using the 6LoWPAN protocol on the Contiki operating system. This protocol allows the use of IPv6 over wireless networks implemented using the IEEE 802.15.4 standard. In the proposed system each sensor node is assigned an IP address.

The transmission of packets using the IPv6 protocol requires an adaptation layer between IPv6 and the IEEE 802.15.4 standard. One of the functions of this adaptation layer is to compress the IP header, as this header is too large for 802.15.4. It also handles the fragmentation and reconstruction of packets because IPv6 supports a maximum transfer unit of 1280 bytes, while the maximum for the IEEE 802.15.4 standard is 127 bytes. It also handles the routing of packets within the local IP network, routing to external IP networks, readings of the status of neighbouring nodes, and support for multicast transmission. In the IEEE 802.15.4 standard, node consumption during transmission varies between 10 and 30 mA, depending on the power level, and communication is performed with a maximum transfer rate of 250 kbit/s. IEEE 802.15.4 uses the ISM band for industrial, scientific, and medical uses: 868 MHz in Europe, 915 MHz in the US, and 2.4 GHz worldwide. However, when designing devices for wireless sensor networks, the 2.4 GHz band is usually chosen since it is not assigned.

**Figure 6:**
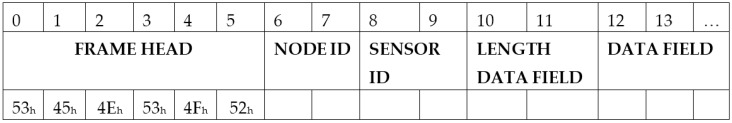
Structure of data packet sent by the sensor nodes.

It should be noted that the sensor network will be implemented following the ad hoc topology. This network topology describes networks as flexible mesh. In these networks all of the nodes provide routing services, so, in addition to functioning as end devices, they also perform packet retransmission multi-hop functions. This way it is possible to transmit packets to nodes with which there is no direct coverage. Thanks to this feature, the network can cover large areas. However, this topology requires routing protocols to calculate the most efficient route to favour lower energy consumption in the nodes. Figure 6 shows the payload of a 6LoWPAN packet structure used in the system for communicating with the nodes. The payload consists of a custom header, IDs for each specific sensor node, the data length and the data itself (a message determining whether a vehicle went in or out). These messages are sent using 6LoWPAN protocol under Contiki.

### 3.3. Detection of the Passage of Vehicles

This section describes the method used to detect the passage of a vehicle on a road into or out of a parking area. The method is designed to detect cars, vans or larger vehicles of two or more axles.

**Figure 7:**
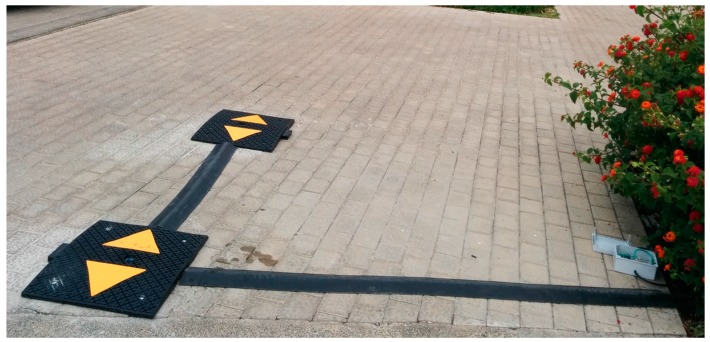
Deployment of the sensors.

The sensor node is connected to two sensors that detect the passage of vehicles. Figure 7 shows the solution proposed using the GP2Y0D02YK0F SHARP (Sharp Corporation, Osaka, Japan) infrared sensor. Each of the two sensors S1 and S2 was placed in a speed bump for protection and as a deterrent to encourage the vehicle’s wheels to pass on each side of the speed bump. The distance, L, between the two bumps was fixed in order to detect vehicles longer than a certain length. The exit and entry monitoring process was carried out by controlling the activation and deactivation event of each of the sensors (On/Off).

Knowing the precise situation of the two sensors and the distance between them, this configuration allows the passage of a vehicle and its direction to be detected, thanks to the order of activation and deactivation of the sensors (see Table 2). When a car is entering or exiting, the system starts from an idle state, in which no sensor is active. Then, an entering or exiting vehicle activates the first sensor, S1 or S2 respectively. As the vehicle continues its movement, the second sensor will be activated: S2 for entering vehicles and S1 for exiting vehicles. In the final stage before returning to an idle state, only one sensor would be active: S2 for entering vehicles and S1 for vehicles moving in the opposite direction.

However, this configuration of sensor pairs is not sufficient to avoid false positives. To decrease the error rate, two timers, T1 and T2, were added to the monitoring process, one for each sensor, to avoid false positives. False positives can be triggered by a moving object; for instance, a person with a shopping trolley, passing through the sensor system, or if a vehicle reverses at the entrance, or if a vehicle uses the lane to perform a manoeuvre to change direction. Therefore, the distance parameters between the sensors and the timers monitoring the time of passage should be set with precision.

**Figure 8:**
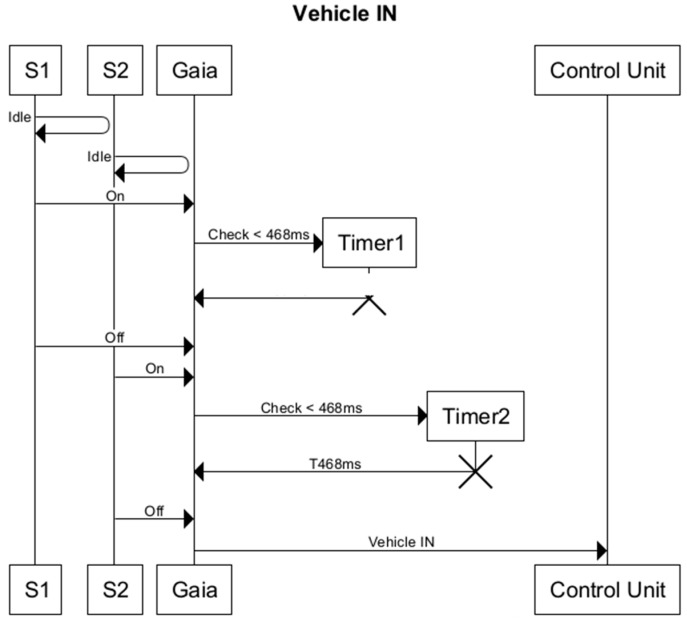
Vehicle entry detection sequence diagram.

The detection algorithm follows the sequence diagram shown in Figure 8. This diagram describes how the T1 and T2 values are associated with each of the sensors to detect different events: passage of a vehicle, vehicle reverses when passing through, or the vehicle is stationary.

As the diagram shows, it is necessary to check that the vehicle has been detected by the two pairs of sensors. This avoids false positives in the event that the vehicle reverses and does not enter, or leaves the parking area.

As the vehicle enters, a reading is taken from both sensors every second. If sensor 1 is activated, timer T1 is initiated to verify that the sensor has been activated by a vehicle and not a person or other object, and once the wait time has passed, a reading is taken from sensor 2.

If sensor 1 is still active but sensor 2 has not been activated, it means that the vehicle has stopped. If, however, sensor 1 is deactivated without sensor 2 having been activated the vehicle has reversed. If sensor 2 has been activated and sensor 1 remains active, timer T2 is initiated to verify that sensor 2 has been activated by a vehicle.

If none of the sensors is then deactivated it may mean that the vehicle is stationary or that a long vehicle is passing. Lastly, a reading of the status of both sensors is taken, in the expectation that sensor 1 has been deactivated before sensor 2, which means that the vehicle has entered the parking area.

To detect a vehicle leaving the parking area, the same process is followed in reverse: first sensor 2 has to be activated and then sensor 1.

**Figure 9:**
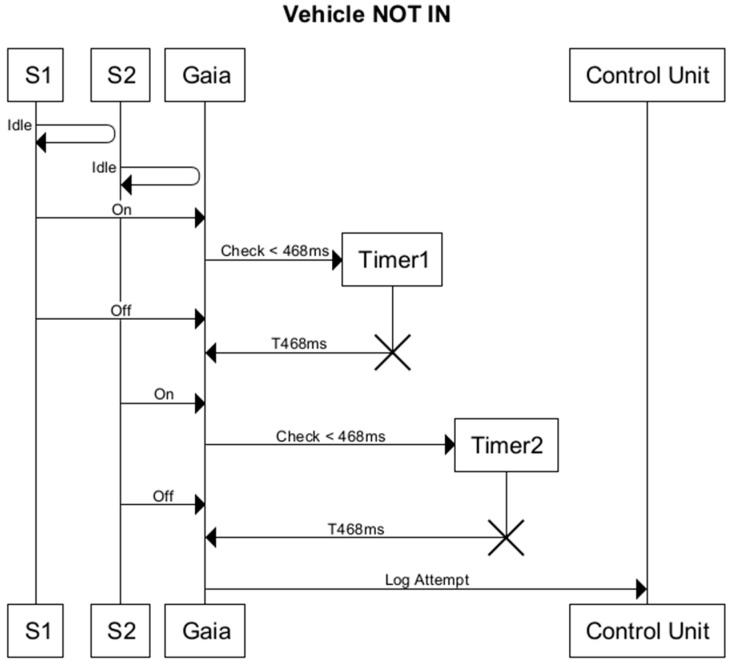
Sequence diagram showing a failed attempt to enter.

In Figure 9 we can see the role that the timer plays in detecting vehicle entry; in this case Timer1 (T1), associated with Sensor1 (S1) has been activated after an Off signal from S1. Therefore, it is not counted as an attempted entry since the vehicle would have had to reach a speed of more than 20 km/h.

**Figure 10:**
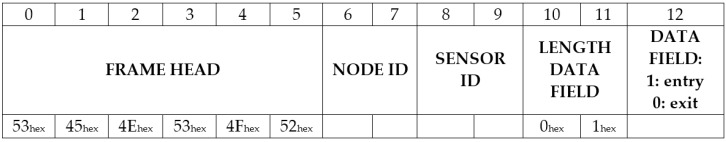
Structure of data packet sent by the sensor nodes to notify vehicle entry or exit.

The “vehicle detection” event, both at the entry and exit control points, is notified by sending a data frame from the sensor node to the gateway node, then on to the data centre. Following the data frame structure described in Section 3.2 and shown in Figure 6 for notification of entry or exit of a vehicle, the data packet sent by the sensor node will be structured as shown in Figure 10: a header of six bytes with the values 53_hex_, 45_hex_, 4E_hex_ ,53_hex_, 4F_hex_, and 52_hex_ (the word SENSOR) followed by the sensor node identifier, the sensor identifier, the number of bytes in the data field of the frame, which in this case is 1 and, finally, the data field containing the value 1_hex_ if notifying vehicle entry or a 0_hex_ if notifying vehicle exit.

## 4. Tests

**Figure 11:**
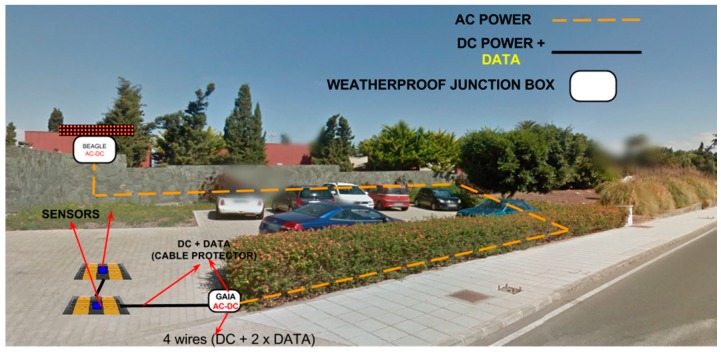
Deployment of prototype sensor system to monitor access to the parking area. The sensors are installed in the speed bumps.

To test the proposed system under real conditions, it was installed in a parking area located on the Tafira Campus of the University of Las Palmas de Gran Canaria. This parking area has a capacity for 10 vehicles and has a single lane that is used both to enter and exit; the setup is shown in Figure 11. The tests were conducted to verify the behaviour of the following aspects of the system: communications system; establishing the configuration parameters of the system for correct detection of the passage of a vehicle (distance between the two speed bumps fitted with a sensor and the time each sensor indicating the presence of a vehicle must be active); and incorrect detection of the passage of a vehicle due to a false positive.

To check the communications system was functioning correctly, an automatic periodic verification process was developed to check the status of each sensor node. Specifically, the sensor nodes were programmed, once they had been activated from the data processing centre, to send the status of their batteries every hour over a period of seven days. It was found that these were correctly identified by their IPv6 address and that the frames were sent correctly without errors and correctly routed through the gateway node to the central data processing system. One hundred sixty-eight packages of 168 were received, with an error rate of 0%. The weather conditions during the test were: clear sky, cloudy sky, and light rain.

Bearing in mind the way that the method of detecting passing vehicles was designed, two values need to be correctly configured for the process to run reliably:

- The distance separating the sensors placed in each speed bump. This is a fixed value that we have called L.
- The time that each sensor of the pair controlled by a node sensor needs to be active, thus indicating the presence of a moving object. This is a value that can be varied by reprogramming the sensor nodes. We have called these values T1 (value of sensor 1 timer) and T2 (value of sensor 2 timer); T1 = T2.

The distance separating the two sensors, each installed in a speed bump, is a fixed value and, for a correct setting, the lengths of the different types of vehicles on the market must be taken into account. For this, we conducted a study of vehicle lengths (see Table 3), concluding that the most appropriate distance is 2.6 m, corresponding to the shortest wheelbase, but longer than the wheelbase of other moving objects, such as a bicycle or a motorcycle.

The time that each sensor must be active indicating the presence of a moving object, T1 for sensor 1 and T2 for sensor 2, depends on the speed of the vehicle in the parking area access lane; the maximum speed allowed in parking areas is 20 km/h. Table 3 shows these values for different types of vehicles travelling at 20 km/h.

False positive situations are impossible to avoid, due to moving objects other than vehicles entering the parking zone, or an intentional improper use of the deployed system. However, these kinds of errors did not appear during the test period, although false negative values were detected. These were caused by vehicles, generally short vehicles, not passing over both sensors situated on the ground, but avoiding one of them when entering or leaving the parking zone. These tests were performed using conventional passenger cars, not other types of vehicles, like trucks or buses, due to parking size restrictions.

## 5. Conclusions

One way to mitigate the negative effects of heavy vehicle traffic in urban and metropolitan areas is the effective management of vehicle parking spaces. Technology has been mooted as the basis for solutions that will have the fastest impact and lowest cost. This article has described a scalable, low-cost, and minimally-intrusive parking space monitoring system, using a wireless sensor network based on IPv6, using the 6LoWPAN protocol, a cloud-based management system and photoelectric sensors. The network sensors are located on speed bumps placed on the way in or out of the parking areas; these sensors are connected to nodes capable of processing the signal sent by the sensors to detect the entry or exit of vehicles into or out of the parking area. These nodes communicate wirelessly with *in situ* signalling devices and gateway units that send the signal to the central management server for processing or distribution via the Internet. The system architecture and the elements that configure it facilitate deployment in parking zones located on public roads. The number of detection monitoring units is low, and the system can, therefore, be scaled up to a city-wide level, monitoring entire areas, as long as all the entry and exit points are controlled.

To check the system, it was tested under real conditions monitoring a car park on a university campus. The tests showed that the system works correctly with an error rate of less than 1%.

For future research, we propose reducing installation impact by incorporating the solar power system and the processing and communication node together with the sensors in the speed bump, as this would further facilitate adoption of this monitoring technology. In addition, tests should be conducted in traffic zones with higher speeds—50 and 60 km/h—in order to be able to implement the system on fast lane exit points and, thus, monitor entire districts of a city.

## Figures and Tables

**Table 1:** Standard sensing modes of the sensor.

Sensing Mode	Advantages	Disadvantages
DIFFUSE 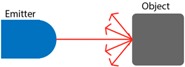	- Ideal for short-range applications. - Does not need a reflector. - Easy installation/alignment.	- The range depends on the characteristics of the object (colour, reflectivity, *etc.*). - A highly reflective background may cause false triggering of the sensor. - Relatively short sensing range.
RETROREFLECTIVE 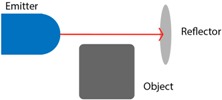	- Moderate sensing range. - Easy to align. - Requires assembly and wiring of only one emitter/receiver unit.	- Shorter sensing range than through-beam. - May detect reflections from shiny objects. - Requires reflector.
THROUGH-Beam 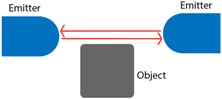	- High margin for contaminated environments. - Longer sensing range than other technologies. - Most reliable sensing mode for highly reflective objects.	- Requires proper alignment. - Not recommended for detection of transparent objects. - Requires space to mount and wire the emitter and receiver individually.

**Table 2:** Map of direction vector control events with two in-line sensors.

Entry		Exit	
S1	S2	S2	S1
Off	Off	Off	Off
On	Off	On	Off
On	On	On	On
Off	On	Off	On
Off	Off	Off	Off

**Table 3:** Values of timers T1 and T2, depending on vehicle type, at a constant speed of 20 km/h.

Vehicle Type	Wheelbase	Value (s)
Bus	15 m	2.7
Articulated bus	18.75 m	3.24
Car	Variable: 6.092 m max. 2.6 m min.	0.468–1.08
Bicycle	1.1 m	0.18

## Abbreviations

The following abbreviations are used in this manuscript:

WHO	World Health Organization
WSN	Wireless Sensor Network
IPv6	Internet Protocol version 6
IPv4	Internet Protocol version 4
UDP	User Datagram Protocol
TCP	Transmission Control Protocol
HTTP	Hypertext Transfer Protocol
6LoWPAN	IPv6 over Low power Wireless Personal Area Networks
RPL	IPv6 Routing Protocol for Low-Power and Lossy Networks
CoAP	Constrained Application Protocol
MVCC	Multiversion Concurrency Control
